# A Gene selection approach based on the fisher linear discriminant and the neighborhood rough set

**DOI:** 10.1080/21655979.2017.1403678

**Published:** 2017-12-19

**Authors:** Lin Sun, Xiaoyu Zhang, Jiucheng Xu, Wei Wang, Ruonan Liu

**Affiliations:** a College of Computer & Information Engineering, Henan Normal University, Xinxiang, Henan, China; b Post-doctoral Mobile Station of Biology, College of Life Science, Henan Normal University, Xinxiang, Henan, China; c Engineering Technology Research Center for Computing Intelligence & Data Mining of Henan Province, Xinxiang, Henan, China

**Keywords:** Gene selection, Fisher linear discriminant, neighborhood rough set, reduction

## Abstract

In recent years, tumor classification based on gene expression profiles has drawn great attention, and related research results have been widely applied to the clinical diagnosis of major gene diseases. These studies are of tremendous importance for accurate cancer diagnosis and subtype recognition. However, the microarray data of gene expression profiles have small samples, high dimensionality, large noise and data redundancy. To further improve the classification performance of microarray data, a gene selection approach based on the Fisher linear discriminant (FLD) and the neighborhood rough set (NRS) is proposed. First, the FLD method is employed to reduce the preliminarily genetic data to obtain features with a strong classification ability, which can form a candidate gene subset. Then, neighborhood precision and neighborhood roughness are defined in a neighborhood decision system, and the calculation approaches for neighborhood dependency and the significance of an attribute are given. A reduction model of neighborhood decision systems is presented. Thus, a gene selection algorithm based on FLD and NRS is proposed. Finally, four public gene datasets are used in the simulation experiments. Experimental results under the SVM classifier demonstrate that the proposed algorithm is effective, and it can select a smaller and more well-classified gene subset, as well as obtain better classification performance.

## Introduction

With the development of gene expression profiles, the analysis and modelling of gene expression profiles has become an important topic in the field of bioinformatics research [1–3]. However, the high dimension of tumor gene expression data, which is often in the thousands or even tens of thousands, increases the learning cost and deteriorates learning performance. This is widely known as the ‘‘Curse of Dimensionality’’, which costs time and reduces the effectiveness of classification when using a classifier to forecast new samples [4,5]. Thus, the dimensionality reduction has been a research hotspot in different fields as an important step in pattern recognition, machine learning and data mining [6-14].

In general, dimensionality reduction algorithms can be categorized as feature extraction and feature selection. Feature extraction constructs a new low-dimensional space out of the original high-dimensional data through projection or transformation, while the aim of feature selection is to reduce the dimensionality of microarray data[15,16] and to enhance classification accuracy [17,18]. The existing feature selection methods can be broadly categorized into the following three classes: filter, wrapper, and hybrid [19]. A good feature selection algorithm should be reasonable and efficient; the algorithm should be able to find a typical genome containing fewer genes [20].

Many scholars have conducted research on gene selection and have generated many results. FLD is a classical technique in pattern recognition; Robert Fisher first developed FLD in 1936 for taxonomic classification [21]. FLD can be used to select the characteristics possessed by classified information, eliminate redundant attributes, and achieve the dimensionality reduction processing of gene data. Since Pawlak in the early 1980s proposed rough set theory, it has been widely used in various fields [22]. However, the classical rough set theory is only applicable to discrete-valued information systems, and it is not suitable for real-valued datasets. To overcome this weakness, Dai and Xu presented a gene selection method based on fuzzy rough sets and a fuzzy gain ratio [23]. Hu et al. proposed a neighborhood rough set model to address both discrete and continuous data sets with a δ-neighborhood parameter, which can maintain the rich information for classifying the data sets [24].

To further improve the classification performance of microarray data, effectively remove the redundant gene, and reduce the computational time complexity of the gene selection algorithm, the FLD method is employed to conduct the preliminary dimensionality reduction for microarray gene data. FLD effectively removes genes that do not contribute to classification. The neighborhood rough set can process continuous data sets and avoid the loss of information caused by discretizing. Then, a new neighborhood dependency and its attribute significance are given, and an attribute reduction method of neighborhood decision systems is presented. A gene selection approach based on FLD and NRS is proposed. A number of simulation experiments were conducted on public gene data sets, and the best parameters were determined according to the experimental results. Therefore, high classification accuracy can be obtained using the selected gene subset under the support vector machine (SVM) classifier [25].

The remainder of this paper is structured as follows: Section 2 introduces related concepts of FLD. An effective and efficient feature selection method based on FLD and NRS is given in Section 3. To evaluate the performance of the proposed algorithm, five related algorithms are employed to compare four public gene expression data sets. The experimental results are described in Section 4. Finally, the conclusion is drawn in Section 5.

## Fisher linear discriminant model

The Fisher linear discriminant is a classical algorithm introduced by Belhumeur in the field of pattern recognition and artificial intelligence [21]. The basic idea of the FLD model is to project the sample onto a straight line by transforming the sample so the projection of the sample can be best divided. That is, the dispersion degree between the transformed sample classes reaches the highest level, and the sample dispersion within the classes reaches the minimum, which increases the distinction among the categories. Therefore, FLD can be used to select the characteristics with the possessed information classified, eliminate redundant attributes, and achieve the processing of dimensionality reduction for gene data. The method is an effective, supervised dimensionality reduction technology. The related concepts of FLD are described as follows.

Let *c* be the number of classes of the sample matrix $X\in R^{d\times n}$, where *n_i_* is the number of samples belonging to the *i*-th class ω*_i_*, and $\sum_{i=1}^{c}n_{i}=n$. The centre point of each sample is $\mu_{i}=\frac{1}{n_{i}}\sum_{x\in \omega_{i}}x$, and the centre point of all samples is $\mu =\frac{1}{n}\sum_{j=1}^{n}x_{j}$, where *x_j_* is the *j*-th sample. The between-class scatter matrix *S_B_* and the within-class matrix *S_W_* can be expressed, respectively, as

(1.1) $$S_{B}=\sum_{i=1}^{c}n_{i}(\mu_{i}-\mu ){\left(\mu_{i}-\mu \right)}^{T},$$

(1.2) $$S_{W}=\sum_{i=1}^{c}\sum_{x\in \omega_{i}}(x-\mu_{i}){\left(x-\mu_{i}\right)}^{T}.$$

On the basis of Formulas (1.1) and (1.2), the between-class scatter *J_B_* and the within-class scatter *J_W_* of the samples after projection are expressed, respectively, as

(1.3) $$J_{B}=\frac{1}{n}W^{T}S_{B}W,$$

(1.4) $$J_{W}=\frac{1}{n}W^{T}S_{W}W.$$

The objective function established by the Fisher discriminant criterion is described by

(1.5) $$\max W\frac{J_{B}}{J_{W}}=\frac{\left|W^{T}S_{B}W\right|}{\left|W^{T}S_{W}W\right|}.$$

If the *k*-th column *w_k_* of *W* is considered, the objective function can be transformed into

(1.6) $$\;\max w_{k}w_{k}{}^{T}S_{B}w_{k}.$$

A Lagrangian equation is established as

(1.7) $$L(w_{k},\lambda )=(1-w_{k}{}^{T}S_{W}w_{k}).$$

Take the derivative of *w_k_*, and make it equal to 0, to obtain the following formula:

(1.8) $$S_{W}{}^{-1}S_{B}=\lambda w_{k}.$$

To maximize the value of $\frac{J_{B}}{J_{W}}$, the projection matrix *W* can be constructed by simply taking the eigenvectors corresponding to the *k* largest eigenvalues.

## Fisher linear discriminant and neighborhood rough set based gene selection method

When using classical rough sets to solve continuous data problems, the data set must be discretized; however, processing the original properties of the data will change, and some useful information will be lost [26]. The neighborhood rough set is proposed to solve the problem where the classical rough set cannot handle the numerical attributes [27,28]. In addition, the effect of the classical neighborhood rough set model is not obvious. Then, to resolve this issue, this paper proposes a feature selection method based on FLD and NRS, which is applied to gene selection of a cancer data set.

There are *N* dimensions in a determined real space *U*. Let Δ = *R^N^*×*R^N^* → *R*. Δ is called as a measure on *R^N^*, and (Δ, *U*) is called as a measure space, when Δ meets the following three conditions:

(1) Δ(*x*_1_, *x*_2_) = Δ(*x*_2_, *x*_1_),
(2) Δ(*x*_1_, *x*_2_) ≥ 0, where the equation holds if and only if *x*_1_ = *x*_2_,
(3) Δ(*x*_1_, *x*_3_) ≤ Δ(*x*_1_, *x*_2_) + Δ(*x*_2_, *x*_3_),

where Δ(*x*_1_, *x*_2_) is a distance function between two elements *x*_1_ and *x*_2_. The distance functions used always include a Manhattan distance function, a Euclidean distance function, and a *p*-normal form distance function. Since the Euclidean distance function can reflect the basic situation of unknown data [29]. the Euclidean distance function is used in this paper. The formula is described as follows:

(2.1) $$\Delta (x_{i},x_{j})=\sqrt{\sum_{k=1}^{N}{\left(f(x_{i},a_{k})-f(x_{j},a_{k})\right)}^{2}}.$$

Let *U* = {*x*_1_, *x*_2_, *x*_3_,…, *x_m_*} be a nonempty finite set on a given real space Ω, then the β-neighborhood of any *x_i_* (1 ≤ *i* ≤ *m*) is defined as

(2.2) $$\beta (x_{i})=\left\{x|x\in U,\Delta (x,x_{i})\;\leq \;\beta ,\beta \;\geq \;0\right\}.$$

Let *U* = {*x*_1_, *x*_2_, *x*_3_,…, *x_m_*} be a nonempty finite set on a given real space Ω, and its neighborhood relationship *N* on the real field Ω is expressed as a binary group *NA* = (*U, N*). For any *X*⊆*U*, the upper approximation and the lower approximations of *X* in a neighborhood approximate space *NA* = (*U, N*) can be defined respectively as

(2.3) $$\bar{N}(X)=\left\{x_{i}|\beta (x_{i})\cap X\neq \phi ,x_{i}\in U\right\},$$

(2.4) $$\underline{N}(X)=\left\{x_{i}|\beta (x_{i})\subseteq X,x_{i}\in U\right\}.$$

The approximate boundary region of *X* is defined as

(2.5) $$BN(X)=\bar{N}(X)-\underline{N}(X).$$

Suppose *NDS* = (*U, A*∪*D*) is a neighborhood decision system, *A* is a conditional attribute set, *D* is a decision attribute, and *U*/*D* = {*X*_1_, *X*_2_, *X*_3_, …, *X_n_*}. For any conditional attribute subset *B*⊆ *A*, the upper approximation and the lower approximation of decision attribute *D* with respect to *B* are expressed, respectively, as

(2.6) $${\bar{N}}_{B}D=\overset{n}{\underset{i=1}{\cup }}{\bar{N}}_{B}X_{i}=\left\{x_{j}|\beta (x_{j})\cap X_{i}\neq \phi ,x_{j}\in U,1\;\leq \;j\;\leq \;\left|U\right|\right\},$$

(2.7) $${\underline{N}}_{B}D=\overset{n}{\underset{i=1}{\cup }}{\underline{N}}_{B}X_{i}=\left\{x_{j}|\beta (x_{j})\subseteq X_{i},x_{j}\in U,1\;\leq \;j\;\leq \;\left|U\right|\right\}.$$

It follows that the boundary region of the decision system can be expressed as

(2.8) $$BN(D)={\bar{N}}_{B}D-{\underline{N}}_{B}D.$$

where $Pos_{B}(D)={\underline{N}}_{B}D$ is a positive domain of the decision system, $Neg_{B}(D)=U-{\bar{N}}_{B}D$ is a negative domain of the decision system.

The existence of the boundary domain causes the uncertainty of the set. Greater uncertainty occurs with larger boundary domain sets. This paper studies the boundary domain of the neighborhood decision system and investigates various uncertainty measures.

The roughness measure, a quantitative index for processing uncertain information by using the rough set theory, is the basis of resource management, system optimization, and many other decision-making problems [30].

Suppose *NDS* = (*U, A*∪*D*) is a neighborhood decision system, *U*/*D* = {*X*_1_, *X*_2_, *X*_3_,…, *X_n_*}. Then for any conditional attribute subset *B*⊆ *A*, the neighborhood precision of *U*/*D* with respect to *B* is described by

(2.9) $$\rho =\frac{\left|{\underset{\_\_}{N}}_{B}D\right|}{\left|{\bar{N}}_{B}D\right|}.$$

The neighborhood roughness of *U*/*D* with respect to *B* is expressed as

(2.10) $$r_{B}(D)=1-\rho =1-\frac{\left|{\underset{\_\_}{N}}_{B}D\right|}{\left|{\bar{N}}_{B}D\right|}=\frac{\left|BN(D)\right|}{\left|{\bar{N}}_{B}D\right|}.$$

Definition 1. Suppose that *NDS* = (*U, A*∪*D*) is a neighborhood decision system and any conditional attribute subset *B*⊆*A.* Then, a dependency of decision attribute *D* with respect to *B* is defined as

(2.11) $$K(B,D)=(1-\rho )\frac{\left|Pos_{B}(D)\right|}{\left|U\right|}=\frac{\left|BN(D)\right|}{\left|U\right|}\frac{\left|{\underset{\_\_}{N}}_{B}D\right|}{\left|{\bar{N}}_{B}D\right|}.$$

Definition 2. Suppose that *NDS* = (*U, A*∪*D*) is a neighborhood decision system, any conditional attribute subset *B*⊆*A*, and *a*∈*B*. Then, an internal significance of attribute *a* with respect to *B* is defined as

(2.12) $$SIG_{inner}\left(a,B,D\right)\;=K\left(B,D\right)－K(B－\left\{a\right\},D).$$

Definition 3. Suppose that *NDS* = (*U, A*∪*D*) is a neighborhood decision system, any conditional attribute subset *B*⊆*A*, and *a*∈*A*−*B.* Then, an external significance of attribute *a* with respect to *B* is defined as

(2.13) $$SIG_{outer}\left(a,B,D\right)\;=K\left(B\cup \left\{a\right\},D\right)－K\left(B,D\right).$$

Definition 4. Suppose that *NDS* = (*U, A*∪*D*) is a neighborhood decision system, any conditional attribute subset *B*⊆*A*, and *a*∈*B*. Then, *B* is a reduction set of *A* if and only if it is satisfied with the following conditions:

(1) *K*(*B, D*) = *K*(*A, D*),
(2) *K*(*B, D*) > *K*(*B*−{*a*}, *D*),

where *K*(*B, D*) is the dependency of the decision attribute *D* with respect to the conditional attribute subset *B*.

Based on the dimensionality reduction technology FLD and the feature reduction of the neighborhood decision system, a feature selection algorithm based on FLD and NRS (FLD-NRS) is designed. The detailed steps are described as follows.

**Input**: A neighborhood decision system *NDS* = (*U, A*∪*D*), and a neighborhood radius β

**Output**: A reduction set *red*

**Step 1**: Calculate a centre point μ*_i_* of the various samples for the conditional attribute set *A* and the centre point μ of all the samples.
**Step 2**: Calculate the within-class scatter matrix *S_W_* and the between-class scatter matrix *S_B_* according to Formulas (1.3) and (1.4).
**Step 3**: Decompose the eigenvalues of $S_{W}{}^{-1}S_{B}$ obtained from Formula (1.8) and sort the eigenvalues with descending order.
**Step 4**: Take the eigenvector corresponding to the first *k* eigenvalues to form the projection matrix *W*.
**Step 5**: Calculate *X*′ = *W^T^X, X′*∈$R^{d^{\prime }}{}^{\times n}$ and obtain the conditional attribute subset *C* after dimensionality reduction that is *NDS* = (*U, C*∪*D*), where *d'* is the number of attributes of *C*, and *n* is the number of samples.
**Step 6**: Let *Ø* → *red*, and *B* = *C*.
**Step 7**: Calculate *SIG_inner_*(*a, B, D*) > 0 with Formula (2.12) for any attribute *a*∈*B* and *a*∉*red*, get the indispensable attribute *a*, and let *red* = *red*∪{*a*}.
**Step 8**: Calculate *SIG_outer_*(*a_k_, red, D*) with Formula (2.13) for any attribute *a_k_*∈*C*−*red*, get the most important attribute *a_k_* according to the size of the order and add it to the reduction set *red* = *red*∪{*a_k_*}.
**Step 9**: Calculate *K*(*red, D*) and *K*(*C, D*) according to Formula (2.11).
**Step 10**: If *K*(*red, D*) ≠ *K*(*C, D*), update the conditional attribute set *C* = *C*−{*a_k_*}, and perform Step 8.
**Step 11**: Output the reduction set *red*.
**Step 12:** End.

For a group of gene expression data, it is assumed that the number of samples is *K*, and the number of attributes is *T*. After the dimensionality reduction according to the FLD algorithm, the *M* genes can be obtained. To select a gene, it is necessary to add $\frac{K}{M}$ samples to the positive domain set, and the neighborhood computational time complexity of the gene data set is *O*(*K*log*K*). Since the computational time complexity of the first gene is *TK*log*K*, and the computational time complexity of the second gene is (*T*−1)(*K*−$\frac{K}{M}$)log(*K*−$\frac{K}{M}$), the computational time complexity of the *M-*th gene is (*T*−*M*+1)log$\frac{K}{M}$log$\frac{K}{M}$. After the above analysis, the worst computational time complexity of the FLD-NRS algorithm is *MTK*log*K*. Due to *M* ≪ *T*, the computational time complexity of the proposed algorithm is less than *O*(*T*^2^*K*log*K*).

## Experimental results and analysis

To verify the effectiveness of the proposed FLD-NRS algorithm, simulation experiments are performed on four public gene expression profile data sets, which include colon, leukaemia, lung, and prostate cancer data downloaded from http://bioinformatics.rutgers.ed/Static/ Supplemens/CompCancer/datasets. The specific description of the datasets is shown in Table 1. The computer system used in this experiment is Windows 7 64-bit operating system, Intel(R) Core(TM) i5-3470 CPU @ 3.20 GHz, Memory (RAM) 4.00 GB. All simulation experiments are implemented in Matlab R2012b (The MathWorks, Inc., 1 Apple Hill Drive Natick, United States).

**Table 1.** Description of the four experimental data sets.

Data set	Feature size	Sample size (normal/tumor)	Class size
Colon	2000	62 (40/20)	2
Leukemia	7129	72 (25/47)	2
Lung	12533	181 (31/150)	2
Prostate	12600	136 (77/59)	2

It is noted that the values of the partial gene columns in the lung and prostate data sets are all zero. Thus, the 121 columns of noise gene data from the lung cancer set and the 394 columns of noise gene data from the prostate set should be eliminated. Finally, the gene number of the lung cancer data set is 12412, and the gene number of the prostate cancer data set is 12206.

In Table 1, the four data sets have two categories, namely, belonging to two classification problems. Taking the colon cancer data set with a high dimension and a small sample as an example, there are 2000 conditional attributes and 62 samples. The number of positive samples is 40, and the number of negative ones is 20. Since the external manifestation of the gene data is a numerical matrix, the model described needs to name the gene dataset as a high-dimensional data matrix, and then the dimensionality needs to be reduced. To ensure that the gene data cannot lose its characteristics, the data of each gene is then marked. That is, the numbers between genes and markers in the data set are corresponding to each other.

In this paper, FLD is employed to preliminary dimensionality reduction, and the neighborhood rough set algorithm is used to further reduce the attributes, which can remove the redundant data of the original data set. The effect of dimensionality reduction then becomes obvious. The neighborhood radius parameter λ is set for each data set, and the lower limit of importance is 0.00001. The FLD-NRS algorithm is used to reduce the attributes for the four data sets respectively in Table 1. The experimental results of the selected gene subsets are shown in Table 2.

**Table 2.** Selected gene subsets of four data sets using FLD-NRS.

Data set	Gene subset after reduction
Colon	{1423, 765, 822, 66, 1870, 590}
Leukemia	{1834, 2354, 2642, 1685, 758}
Lung	{2549, 7200, 6139}
Prostate	{8986, 11052, 6392, 4050}

**Figure 1.**
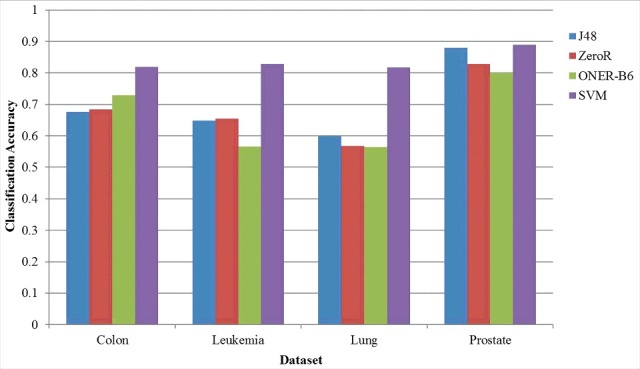
Classification accuracy of the four data sets under different classifiers.

To verify the classification performance of the selected gene subsets, four classifiers are employed to do this experiment on each data set. The results are indicated in Figure 1.

According to Fig. 1, SVM has the best classification performance on the four data sets when compared with the other three. Then, to verify the validity of the proposed algorithm for selecting a gene subset with strong classification, the classification accuracy of the gene subset after reduction is evaluated on SVM. The FLD-NRS algorithm is compared with the other three related algorithms on four gene data sets, where the original data processing (ODP) algorithm is used to classify the original data set directly. The Lasso[31,32] algorithm is a feature selection method by coefficient compression estimation, and the NRS [24]. algorithm is a feature selection method using the neighborhood rough set theory. The experimental results are illustrated in Table 3, where *m* describes the gene number after gene selection, and *Acc* describes the optimal classification accuracy. Meanwhile, the time complexities of these algorithms are given in Table 3.

**Table 3.** Selected gene number and classification accuracy of the four algorithms on different data sets.

	ODP		Lasso		NRS		FLD-NRS	
Data set	*m*	*Acc*	*M*	*Acc*	*m*	*Acc*	*m*	*Acc*
Colon	2000	0.811	5	0.887	4	0.611	6	0.880
Leukemia	7129	0.944	23	0.986	5	0.645	5	0.828
Lung	12412	0.903	8	0.995	3	0.641	3	0.889
Prostate	12206	0.619	63	0.961	4	0.647	4	0.800
Time complexity	—		*O*(*PT*^3^)		*O*(*T*^2^*K*log*K*)		*O*(*MTK*log*K*)	

It can be seen from Table 3 that, although the classification accuracy of the leukemia data set is 94.4% with the ODP algorithm, the original data is directly classified by using ODP, and the size of the selected gene subset is very large. The NRS algorithm can effectively remove irrelevant genes to obtain a smaller gene subset. However, some genes with strong classifications have been removed. This leads to the lower classification accuracy of the selected gene subset. For example, the classification accuracy of leukemia with NRS is reduced to 64.5%. In the process of gene selection, it is known that the scale of the selected gene subset and its classification accuracy are two important aspects. The FLD-NRS algorithm presented in this paper can select a smaller gene subset, and the classification accuracy has clearly been improved. For the colon data set, our algorithm can also select fewer genes with higher classification accuracy than the other two algorithms. For the leukemia and lung data sets, although our accuracy is lower than those of the ODP and Lasso algorithms, the selected gene subset is much smaller than those of the above two algorithms. Meanwhile, for the prostate data set, the classification accuracy with FLD-NRS is higher than those of the ODP and NRS algorithms, and the selected gene number is smaller than those of the ODP and Lasso algorithms. These results prove the effectiveness of the proposed algorithm for gene selection.

To further investigate the performance of the proposed algorithm, the FLD-NRS algorithm is compared with two random forest algorithms, where RF represents the classical random forest algorithm [33]. and SNRRF [34]. represents an improved random forest algorithm. The time complexity of the random forest algorithms can be approximated as *O*(*kTK*(log*K*)^2^), where *k* is the number of random classifiers in a random forest. The experimental results are shown in Table 4. The time complexities of these algorithms can be found in Table 4.

**Table 4.** Selected gene number and classification accuracy of three algorithms on different data sets.

	RF		SNRRF		FLD-NRS	
Data set	*m*	*Acc*	*m*	*Acc*	*m*	*Acc*
Colon	2000	0.848	72	0.875	6	0.880
Leukemia	7129	0.902	26	0.948	5	0.828
Lung	2880	0.864	10	0.899	3	0.889
Prostate	12600	0.925	49	0.931	4	0.800
Time complexity	*O*(*kTK*(log*K*)^2^)		*O*(*kTK*(log*K*)^2^)		*O*(*MTK*log*K*)	

According to Table 4, the classification accuracy of the colon data set with the FLD-NRS algorithm is 88%, which is higher than those of the RF and SNRRF algorithms. For the lung data set, the accuracy of the proposed algorithm is 88.9%, which is basically equivalent to those of the two random forest algorithms, but the selected gene number is very small. These results demonstrate the validity of the proposed algorithm. However, for the leukemia and prostate data sets, the classification accuracy of this algorithm is slightly lower than those of the two random forest algorithms. These results explain that when using FLD to filter irrelevant genes, the genes with large influence on classification are mistakenly filtered out; therefore, the classification accuracy will be affected and reduced.

Through the time complexity analyses presented in Tables 3 and 4, it is obvious that the Lasso algorithm costs significantly more time, which is higher than those of the other four algorithms; although, the classification accuracy of the selected gene subset is high. The gene number of the original data set is usually much larger than that of the selected gene subset, so the time complexity of the proposed algorithm is obviously lower than those of the other five algorithms.

The above experimental results show that the FLD-NRS algorithm can solve the high-dimensional and high-redundancy problem of gene expression profile data well. The selected gene subset is smaller, and the dimensionality reduction effect is obvious. Hence, the FLD-NRS algorithm is superior to the other four algorithms mentioned in this paper under the overall situation of the three indicators, including selected gene number, classification accuracy, and computational time complexity. Therefore, the FLD-NRS algorithm can accomplish dimensionality reduction processing well, and the selected gene subset has strong classification abilities.

## Conclusion

The challenge of selecting genes with important classification information from tens of thousands of gene expression profiles is an important problem in the field of bioinformatics. In this paper, a genetic selection method based on the FLD and NRS is proposed in view of poor stability, large feature subset size, and time-consuming calculations of various gene selection algorithms. The FLD approach is applied into the preliminary dimensionality reduction of gene data to obtain the candidate gene subset. A novel feature reduction algorithm in neighborhood decision systems is proposed to optimize the features after dimensionality reduction. Then, a gene subset with strong classification ability is selected. The experimental results all show that the FLD-SNR algorithm can select a gene subset with smaller scale and stronger classification ability. The proposed algorithm is of great practical significance for the future study of cancer clinical diagnosis.
